# Interleukin 41 As A Potential Predictor of Bio-Therapy Efficacy In Patients With Rheumatoid Arthritis: A Prospective Observational Study

**DOI:** 10.7150/ijms.98752

**Published:** 2024-09-30

**Authors:** Jasmina Jocić, Aleksandra Tomić Lučić, Ivan Jovanović, Milena Jurišević, Isidora Stanisavljević, Bojana Stamenković, Slađana Pavlović

**Affiliations:** 1 Institute for Treatment and Rehabilitation, Niška Banja, Serbia.; 2 Faculty of Medical Sciences, Department of Internal Medicine, University of Kragujevac, Kragujevac, Serbia.; 3 Faculty of Medical Sciences, Center for Molecular Medicine and Stem Cell Research, University of Kragujevac, Kragujevac, Serbia.; 4 Faculty of Medical Sciences, Department of Clinical Pharmacy, University of Kragujevac, Kragujevac, Serbia.

**Keywords:** cytokines, IL-41, rheumatoid arthritis, biomarker

## Abstract

**Introduction:** A novel immunomodulatory cytokine IL-41 is associated with the pathogenesis of Graves disease, Kawasaki disease, gout, psoriatic arthritis, and rheumatoid arthritis (RA). We aimed to evaluate serum IL-41 level as a biomarker of the RA and disease activity treatment efficacy and patient responses. We also wanted to determine eventual potential predictors of IL-41 concentrations.

**Methods and analysis:** This observational clinical trial will enrol 189 patients rheumatology clinics of the Clinical Center Kragujevac, Serbia. Participants will be divided into three groups: patients on methotrexate monotherapy (MTX), (n=31), those treated with combined therapy of MTX plus TNF inhibitors (TNFi) (n=70), and patients treated with monotherapy with IL-6 inhibitor tocilizumab (TCZ) (n=43). Newly diagnosed RA or patients who for some reason were excluded from the DMARDs for a minimum of 3 months were considered as the control group (n=45).

**Results:** TCZ reduced the IL-41 level the most. All treatment options significantly reduced clinical signs, symptoms and the scores of disease activity composite indices, TCZ the most. The only statistically significant predictor of higher IL-41 values was smoking.

**Conclusion:** IL-41 may be a new potential biomarker that can help physicians evaluate treatment efficacy and predict patient responses. Smoking status is associated with the higher concentration of IL- 41 and clinical presentation of patients with RA.

## 1. Introduction

Rheumatoid arthritis (RA) is a chronic, systemic inflammatory autoimmune disease that causes progressive joint damage and consequently disability 1. In addition, RA is associated with extraarticular tissue damage in the heart, lungs, kidneys, blood vessels, digestive system, eyes, skin and nervous system 2,3. Genetic and environmental factors contribute to the pathogenesis of RA and the immunological pathway may precede years before the joint inflammation. When these factors interact, autoantigens are modified and the immune system becomes unable to recognize some proteins as self-structure, creates autoantibodies and accidentally targets own tissue 4.

Two autoantibodies are important serological markers for the diagnosis and treatment evaluation of RA: rheumatoid factor (RF) and anti-cyclic citrullinated peptide (CCP) antibody (ACPA). They form complexes with IgG and citrullinated proteins and migrate to synovial fluid. ACPA - citrullinated protein complex triggers macrophages to release inflammatory cytokines such as tumor necrosis factor (TNF) and interleukin (IL)-6. In addition, Th 17 cells secrete IL-17 and these proinflammatory cytokines all together trigger other cells to secrete additional proinflammatory mediators: IL‑8, IL‑12/IL‑23, IL‑17, IL‑18, IL‑32 and interferon‑γ (IFN‑γ) that ultimately damage joints 5. New therapeutic options of biological disease-modifying antirheumatic drugs (bDMARDs) targets some of these molecules and are effective in combination with conventional DMARDs in moderate to severe RA 6.

A novel immunomodulatory cytokine IL-41 (synonyms: IL-41, Cometin, Subfatin, Meteorin (Metrn)-like, or Meteorin-β) is decreased in inflammatory bowel disease 7, Graves' disease 8 and is elevated in Kawasaki disease 9, gout 10, psoriatic arthritis 11 and RA 12,13. These findings sugest the potential role of IL-41 in abnormal immune responses. An increase of IL-41 in the serum is also associated with the disease activity of RA 13. Although the role of IL-41 in the pathogenesis of RA is still not fully understood, it is believed that abnormal expression of IL-41 inhibits the differentiation of osteoblasts in patients with RA, thus affecting the progression of the disease 12. Previous studies have shown that immune cells produce IL-4 and IL-17A, which stimulate macrophages to produce IL-41, while IFN-γ inhibits IL-41 production. This suggests that IL-41 may be involved in Th1, Th2, and Th17 immune responses 15.

Our study aims to evaluate the relationship between IL-41 levels and disease activity and treatment efficacy. The study also aims to determine potential predictors of IL-41 concentrations.

## 2. Materials and Methods

### 2.1. Study design and Study population

This study is designed as a prospective observational analytical study. A total of 189 patients with RA, 35 men and 154 women who met the classification criteria of the ACR/EULAR 2010 14 and signed an informed consent were included in the study. All patients were between 21 and 81 years old (57.9±12.6). The study did not include patients with acute or chronic inflammatory, infectious, hematological, endocrine and psychiatric diseases, pregnancy, postpartum, malignancy, renal failure and liver failure. Patients were divided according to the type of therapy into three groups: patients on methotrexate monotherapy (MTX), (n=31), those treated with combined therapy of MTX plus TNFi, (n= 70), and patients treated with monotherapy with IL-6 inhibitor tocilizumab (TCZ) (n=43). Newly diagnosed RA or patients who for some reason were excluded from the DMARDs for a minimum of 3 months were considered as the control group.

### 2.2. Variables measured in the study

The concentration of IL-41 was tested as a potential biomarker of disease activity, as well as a potential biomarker of therapeutic response. We compared the concentration of IL-41 as a potential biomarker of disease activity as well as potential biomarker of therapeutic response. We corelated the concentration IL-41 with established biomarkers of disease activity: clinical (tender joint count - TJC; swollen joint count - SJC, pain degree on a Visual Analogue Scale - VAS); laboratory (erythrocyte sedimentation rate - ESR; C reactive protein - CRP) and composite indices of disease activity: disease activity score in 28 joints based on erythrocyte sedimentation rate - DAS28, clinical disease activity score - CDAI and simplified disease activity score - SDAI. We assessed possible association the concentration of IL-17 and IL-23 with concentrations of IL-41. All variables were included in linear regression analysis to confirm their possible predictive value for IL-41 concentrations.

### 2.3. Serum samples

Determination of biochemical analyzes was performed in the Central Biochemical Laboratory of the University Clinical Center Kragujevac using standard methods, using the Beckman Coulter AU 400 Unicel DXC 800 Synchron Clinical System: CRP (C-reactive protein) was determined by an immunoturbidimetric test for the quantitative determination of CRP in human serum and plasma, expressed in mg/L (reference value 0-5). ESR (erythrocyte sedimentation rate) was determined by the Westergreen method, expressed in mm/h (reference value for men <50 years: ≤15 mm/h; women <50 years: ≤ 20 mm/h; men >50 years ≤20 mm/h, women >50 years ≤30 mm/h). The levels of RF (IGM rheumatoid factor) were measured by the immunoturbidimetry method, the result was considered positive when the obtained value was above the reference value <14 IU/ml. ACPA (anti-citrullinated protein/peptide antibody) was performed using the ECLIA method on the Cobas C 601 device (reference values for both men and women 7-17 IU/ml).

### 2.4. Measurements of serum cytokines levels

Patient sera were collected by venipuncture. After centrifugation, the serum was separated into test tubes and stored at -20°C until thawed for analysis. Levels of interleukin-41 (IL-41), interleukin-17 (IL-17) and interleukin-23 (IL-23) were measured in serum with highly sensitive enzyme-linked immunosorbent assay (ELISA) kits (Human Meteorin-like/METRNL DuoSet ELISA Bio-Techne includes R&D Systems, Human IL-23 DuoSet ELISA, Human IL-17 DuoSet ELISA, R&D Systems) specific for human cytokines according to the manufacturer's instruction. Every sample under-went duplicate testing.

### 2.5. Statistical Analysis

We used descriptive statistical methods, methods for statistical hypothesis testing, association analysis methods and relationship modeling methods for outcomes and potential predictors. Depending on the type of variables and the normality of the distribution, description data is presented as n (%), arithmetic mean ± standard deviation or median (min-max). Among the methods for testing statistical hypotheses, we used: chi-square test, Fisher's exact test probabilities, ANOVA, Kruskal-Wallis test. Tukey was used for multiple comparisons of data post hoc test or Bonferroni procedure. Among the methods for analyzing the association of cytokines, we used Spearman's correlation coefficient. For modeling the relationship of the dependent variable (IL-41) with potential predictors, we used linear correlation. IL-41 has a normal distribution and was analyzed as such, unlike IL-17 and IL-23 where robust regression (chi square test) was used; variables are described in the table as median (minimum-maximum range). Multivariate regression models included predictors from univariate analyses which were considered statistically significant at the 0.05 significance level. Statistical hypotheses were tested at a statistical significance level (alpha level) of 0.05. All data were processed in IBM SPSS Statistics 22 (SPSS Inc., Chicago, IL, USA) software package or the R programming environment (R Core Team, 2019).

## 3. Results

### 3.1. Characteristics of the study population

Laboratory parameters and composite indices of disease activity are shown in Table 1. We found statistically significant differences between the groups in age (p=0.014) and pain measured by visual analog scale (PS-VAS, p<0.001). DMARD naive patients were the oldest group 67.0±13.5 vs 55.1±13.2 (TCZ) vs 61.35±10.9 (MTX+TNFi) vs 61.6±13.5 (MTX). Patients on TCZ and MTX+TNFi had the lowest PS-VAS scores. All activity parameters were significantly lower in each of the three treatment groups compared to the control group at the same level of probability p<0.001. There was no statistically significant difference in the values of RF and ACPA relation to the control group (RF: p=0.326; ACPA: p=0.607).

### 3.2. Cytokine profile

Treatment options affect cytokine profile (Table 2). TCZ reduces IL-41 the most. MTX also reduces IL-41 but without significance when compared to control group. We noticed the lowest level of IL-23 in MTX + TNFi group. Interestingly, none of the therapeutic choices didn't show any influence on the IL-17 concentrations. Paradoxally, the control group had a lowest level of IL-17 which was statistically significant compared to all 3 therapeutic groups. Only MTX + TNFi significantly reduced IL-23 concentration. All differences between the groups were statistically significant (Table 2).

### 3.3. Linear regression analysis

In the multivariate linear regression model with IL-41 values as a dependent variable, we included variables that were statistically significant at the 0.05 significance level in the univariate models. Due to multicollinearity with the variable medications, the variables DAS28 and CDAI were not included in the multivariate model. The only statistically significant predictor of higher IL-41 values was smoking. Other variables were not predictive for of the higher IL-41 concentration (Table 3).

## 4. Discussion

To our knowledge, this is the first study that attempted to determine the potential role of IL-41 as a biomarker of disease activity and its role in evaluating outcomes of different treatment options for RA. We assessed the efficacy of MTX, MTX plus TNFi and TCZ by their impact on IL-41 concentration. We found that all three types of treatment reduce the level of IL-41; treatments with MTX plus TNFi and TCZ reach statistical significance. All drugs significantly reduce clinical, biochemical and composite indices of disease activity, TCZ the most. Only the combination of MTX plus TNFi significantly reduces IL-23 concentration. The only statistically significant predictor of higher IL-41 values was smoking.

Data on the effects of different treatments on IL-41 concentrations are scarce. IL-41 stands for a novel immunomodulatory cytokine associated with the pathogenesis of various inflammatory diseases in both animal and human models. Ushach and colleagues showed that IL-41 participates in inflammatory response in mice 15. Serum concentrations of IL-41 in patients suffering from RA are elevated compared to healthy controls and correlate with DAS28, CRP and ESR 12 confirming its association with inflammation and disease activity in RA patients 13. We found significant differences between groups in the values of disease activity indices DAS28-ESR, CDAI and SDAI. Similar association was found for the concentration of IL-41 suggesting the possibility of its influence on inflammation and disease activity in RA patients.

Treatment guidelines include conventional DMARDs, biological DMARDs, or their combination. Recommendations are to initially start with methotrexate with folic acid and then consider various DMARDs if methotrexate is not effective enough 16. A network meta-analysis by Hazlewood and colleagues demonstrated the superiority of several treatment combinations to methotrexate alone, including methotrexate + most biologics with moderate to high-quality evidence 17. Scientists debate if it is better to immediately start with aggressive, biological DMARDs alone or in combination with methotrexate, rather than methotrexate alone. This approach would increase the number of patients who achieve and remain in remission 18. We showed that DMARDs and combinations significantly reduce IL-41 serum concentrations. The bDMARDs may have a stronger impact on immune response and overall inflammation in RA due to the greater effect on IL-41 levels compared to methotrexate.

The possible underlying mechanisms of different IL-41 levels after treatments with different DMARDs included in this study may be related to cytokines that influence its production. Methotrexate alone yielded the highest level of IL-17. The cytokines TNF, IL-17A and IL-17F are unable to efficiently stimulate synovial fibroblasts to produce IL-41 when used separately, one at a time; but IL-17A or IL-17F synergistically with TNF increase IL-41 production 11. If the concentrations of any of these three cytokines decrease, IL-41 also decreases. When comparing responders to non-responders, after at least 6 months of any 3 therapeutic modalities used, IL-17A levels were even higher than in the control group. 19. This insufficient effect on IL-17 production and the presence of the non-responders in our study population, may be the reasons we found methotrexate to have the lowest impact on IL-41 and the reasons for some scientists suggest methotrexate alone may only be suitable for the early stages of RA 6.

In our population, TNFi together with MTX reduced IL-41 more than methotrexate alone, probably due to TNF inhibition, but IL-17 concentration was higher compared to DMARDs naïve patients. This confirms the importance of the synergistic effect of both cytokines - IL-17 and TNF - on IL-41 concentration. An additional explanation may be that some TNFs reduce IL-17 production by 25% while MTX has a modest effect 20. MTX alone has a mild effect on IL-23/IL-17 axis in our study. Our MTX group of patients had the highest level of IL-23. This is probable due to the high percentage of non-responders to MTX in our study population, given that the IL-23 may be the best cytokine to differentiate between responders and non-responders to MTX. If one of the main drivers of inflammation is IL-23, MTX will be ineffective 21. Although some scientists suggested that IL-23/IL-17 axis plays important role in RA pathogenesis and should be considered for the development of targeted therapies 22, our results indicate that IL-41 may be even more important for RA pathogenesis and treatment outcomes. Accordingly, the synergistic effect of IL-17 and TNF on IL-41 concentration indirectly affects RA.

TCZ significantly decreased IL-41 compared to the DMARD naïve group of patients in our study. It even yielded the lowest IL-41 concentration. However, IL-17 concentration was higher than in the control group. Both IL-17 and TNF activate synovial fibroblasts to produce IL-6 23, but IL-6 is unable to bind to its receptors because TCZ blocks them. TCZ also decreases TNF concentrations 24 and thus likely reduces IL-41 in our study population, regardless of higher IL-17.

We found lower IL-41 concentrations in tocilizumab and MTX +TNFi groups and lower disease activity parameters in the same groups. This confirms that clinical presentation of the patient is more associated with the IL-41 than IL-17 concentrations and that IL-41 may be one of the key cytokines in the pathogenesis of RA with more important role than IL-17. Our results also suggest that IL-41 may be useful parameters of disease activity and as a predictor of treatment response since patients with the best clinical outcomes had the lowest IL-41 levels.

As a second aim, we wanted to discover factors associated with IL-41 concentration. We found that smoking status was the only predictor that remained statistically significant in the multivariate regression model after adjustment. We also confirmed that tocilizumab and MTX+TNFi therapies may be factors affecting IL-41 levels, but this association did not reach significance in multivariate analysis. Although we showed that these treatments decrease IL-41 levels, their potential predictive values have yet to be confirmed in future studies. The most important genetic factor for RA development is shared epitope alleles, but this doesn't mean clinical development of the disease 25. As RA is a multifactorial disease determined by genetic and environmental factors, smoking status is the most important in the latter group 26. Smoking is associated with the production of the RF and ACPA autoantibodies 27, and the risk of seropositive RA development is much higher 28. It is a proinflammatory process that activates epithelial cells to produce cytokines towards Th1 and Th17 CD4 T-cells 29. It also increases the level of TNF 30 and may be the reason for the discontinuation of the treatment with TNF inhibitors 31. The fact that cigarette smoking increases both IL-17 and TNF may explain its predictor role for increased IL-41 concentration. The data on the association between smoking status, level of IL-41 and antibodies are still inconsistent. The evidence suggests that smoking may be more strongly linked to RF than anti-citrullinated protein antibody (ACPA) 27. Additionally, smoking may have a dual effect. It can alter the autoantigens that the immune system responds to, and it can also impact the immune system itself 27. Given that smoking can increase the citrullization of antigens and the concentration of IL-41^32^, it would be expected that a decrease in IL-41 would indirectly lead to a decrease in ACPA. However, we did not find a connection between the level of IL-41 and ACPA. Similarly, Zhang et al 13 found no correlation between IL-41 and ACPA. Given that, among other autoantibodies, ACPA and RF often occur together in RA, it is unclear whether smoking status is specifically associated with some autoantibodies rather than others 27. This non-specificity may explain the inconsistency in results, and it seems that further studies are needed to elucidate the mechanisms involved.

Our study is limited by the ratio of subjects between groups. We included a total of 154 patients who were unevenly distributed. This may have prevented us from detecting some of the associations between groups, but we believe that the data we analyzed are sufficient to draw valid conclusions.

## 5. Conclusion

According to our results, IL-41 may be a new potential biomarker that can help physicians to evaluate disease activity and thus treatment efficacy and to predict patient responses. Our results also imply its involvement in the pathogenesis of RA and its potential as a therapeutic target for RA. Smoking status is associated with higher concentration of IL-41.

## Figures and Tables

**Figure 1:**
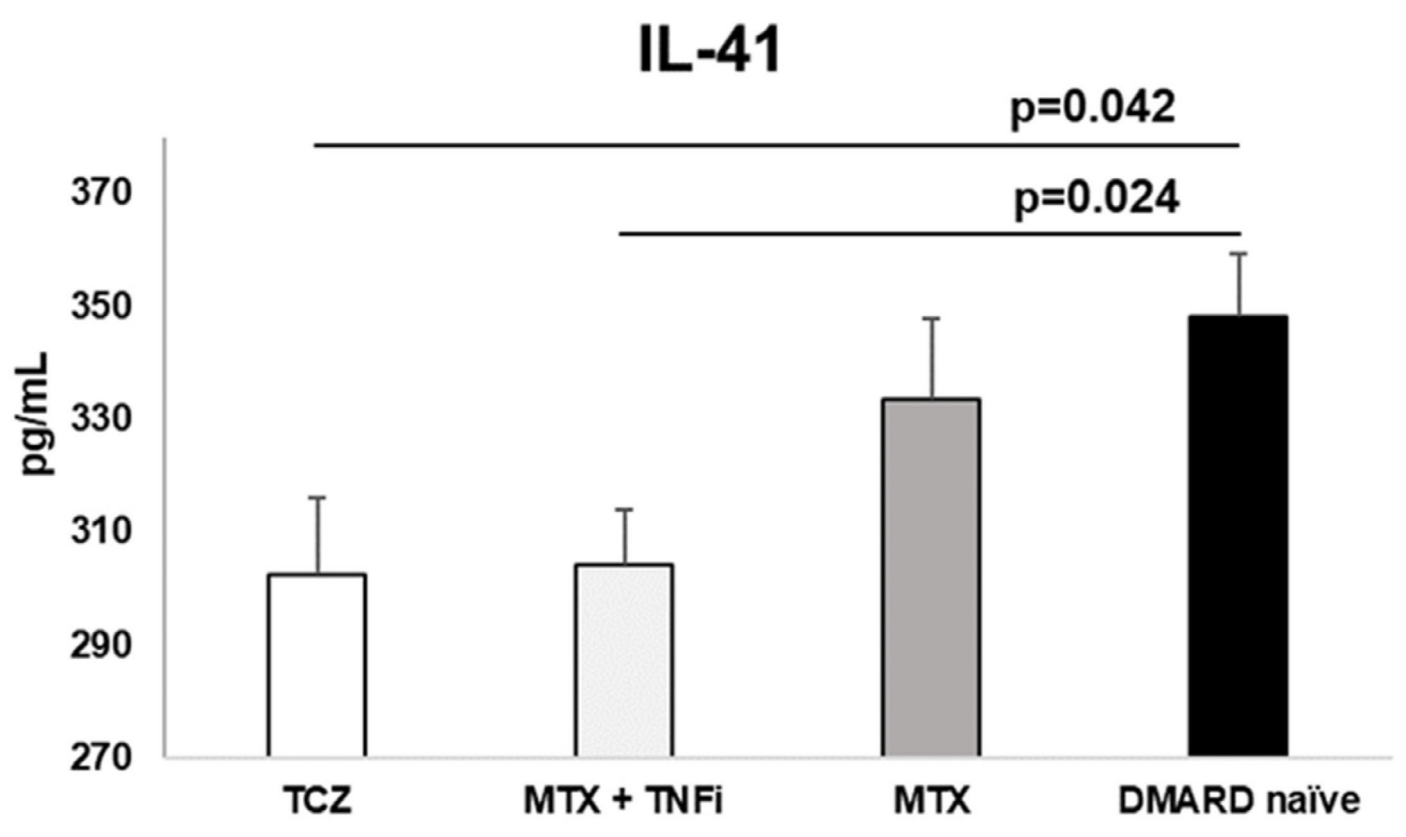
Treatment with TCZ and MTX + TNFi modified the serum levels of IL-41 in RA patients. IL-41 was significantly lower in TCZ and MTX + TNFi treated groups compared to control group. Data is presented as arithmetic mean ± standard error.

**Table 1:** Laboratory parameters and composite indices of disease activity of the study population in relation to the therapeutic group

	Established RA (n=144)			DMARD naive	
Variables	TCZ (n=43)	MTX+TNFi (n=70)	MTX (n=31)	Control group (n=45)	p-value
ESR as mm/hour, median (range)	5.0 (1.0- 32.0)	17.0 (2.0- 85.0)	22.0 (2.0-74.0)	30.0 (6.0-135.0)	< 0.001
CRP as mg/L, median (range)	0.4 (0.0- 8.0)	2.9 (0.0-40.1)	3.0 (0.0-91.0)	8.2 (0.0-101.8)	< 0.001
TJC, median (range)	3.0 (0.0-10.0)	3.0 (0.0-13.0)	5.0 (0.0-12.0)	6.0 (0.0-18.0)	<0.001
SJC, median (range)	0.0 (0.0-6.0)	2.0 (0.0-9.0)	4.0 (0.0-10.0)	5.5 (0.0-11.0)	<0.001
PS-VAS, median (range)	30.0 (10.0- 75.0)	30.0 (0.0-80.0)	40.0 (10.0-80.0)	60.0 (0.0-100.0)	<0.001
DAS28-ESR (mean±SD)	2.7±0.7	3.6±1.1	4.4±1.3	5.0±1.5	<0.001
CDAI, median (range)	10.5 (6.0-26.0)	11.5 (1.5-42.0)	16.0 (2.0-38.0)	24.0 (2.0-45.8)	<0.001
SDAI, median (range)	9.9 (3.0-27.0)	12.0 (2.0-35.6)	16.3 (2.0-45.1)	24.1 (2.0-47.4)	<0.001

*Abbreviations: RA:* rheumatoid arthritis, *ESR:* erythrocyte sedimentation rate; CRP: C-reaction protein; *TJC*: tender joint count; *SJC*: swollen joint count; *PS-VAS:* pain score measured by a visual analogue scale, *DAS28-ESR:* disease activity score in 28 joints based on erythrocyte sedimentation rate, *CDAI:* clinical disease activity score*, SDAI:* simplified disease activity score.

**Table 2:** Cytokine values in relation to treatment options

Cytokine profile/medications	TCZ (n=43)	MTX + TNFi (n=70)	MTX (n=31)	DMARD naïve (n=45)	Overall p value
IL-41 (ng/mL)	302.6±87.3 *p=0.042	304.1±81.4 †p=0.024	333.4±79.5 p=0.858	348.3± 72.8	**0.013**
IL-23 (pg/mL)	28.9 (11.2-521.9) p=0.536	23.5 (1.2-275.6) ‡p=0.032	53.7 (14.5-403.2) p=1.000	40.2 (10.1-531.1)	**0.007**
IL-17 (pg/mL)	71.0 (15.4-110.1) ¥p=0.003	65.9 (21.0-120.3) ¥p=0.048	65.7 (33.4-126.3) ¥p=0.001	44.6 (20.4-197.9)	**<0.001**

Values for IL-41 in relation to the groups do not deviate from normality, so they were analyzed by ANOVA. Values for IL-23 and IL-17 deviated from normal distribution and were reported as median (range) and analyzed as Kruskal-Wallis test. *Refers to statistically significant differences analyzed via ANOVA (p=0.042) between the group on tocilizumab therapy and DMARD naive patients. †Refers to statistically significant differences analyzed via ANOVA (p=0.024) between the MTX + TNFi therapy group and DMARD naïve patients. ‡ Refers to statistically significant differences analyzed via Kruskal-Wallis test (p=0.032) between the MTX + TNFi therapy group and DMARD naïve patients. ¥ All differences between the groups were statistically significant.

**Table 3:** Linear regression with IL-41 values as dependent variable

Variable	Univariate		Multivariate	
	B	p-value	B	p-value
Medications				
MTX	-14.850	0.431	-5,121	0,802
MTX+TNFi	-44.148	0.005	-36,35	0,051
TCZ	-45.635	0.009	-30,59	0,18
Sex (F/M)	-26.710	0.083		
Age	0.502	0.295		
RF	-7.660	0.730		
ACPA	-0.953	0.962		
ESR	0.758	0.005	0.440	0.180
CRP	0.541	0.176		
SJC	5.275	0.009	1.826	0.455
Global health on VAS (mm)	0.388	0.187		
HAQ-DI	-12.350	0.221		
Radiographic erosions	-4.561	0.512		
Smoking status	33.073	0.012	33.635	**0.009**
Pain detect test	-4.008	0.005		
DAS28-ESR	14.717	<0.001		
CDAI	1.494	0.017		

*Abbreviations: MTX:* methotrexate*, MTX+TNFi:* methotrexate and Tumor Necrosis Factor Inhibitors,* TCZ:* tocilizumab,* RF:* rheumatoid factor,* ACPA:* anti-cyclic citrullinated peptide antibodies,* ESR:* erythrocyte sedimentation rate,* CRP:* C-reaction protein,* SJC:* swollen joint count,* Global health on VAS:* pain score measured by a visual analogue scale,* HAQ-DI:* Health Assessment Questionnaire Disability Index,* DAS28-ESR:* disease activity score in 28 joints based on erythrocyte sedimentation rate,* CDAI:* clinical disease activity score.

## References

[1] Romão VC, Fonseca JE (2021). Etiology and Risk Factors for Rheumatoid Arthritis: A State-of-the-Art Review. Front Med.

[2] Ciobanu DA, Poenariu IS, Crînguș LI, Vreju FA, Turcu-Stiolica A, Tica AA (2020). JAK/STAT pathway in pathology of rheumatoid arthritis (Review). Exp Ther Med.

[3] Wu D, Luo Y, Li T, Zhao X, Lv T, Fang G (2022). Systemic complications of rheumatoid arthritis: Focus on pathogenesis and treatment. Front Immunol.

[4] Radu AF, Bungau SG (2021). Management of Rheumatoid Arthritis: An Overview. Cells.

[5] Kondo N, Kuroda T, Kobayashi D (2021). Cytokine Networks in the Pathogenesis of Rheumatoid Arthritis. Int J Mol Sci.

[6] Prasad P, Verma S, Surbhi, Ganguly NK, Chaturvedi V, Mittal SA (2023). Rheumatoid arthritis: advances in treatment strategies. Mol Cell Biochem.

[7] Gholamrezayi A, Mohamadinarab M, Rahbarinejad P, Fallah S, Barez SR, Setayesh L (2020). Characterization of the serum levels of Meteorin-like in patients with inflammatory bowel disease and its association with inflammatory cytokines. Lipids Health Dis.

[8] Gong L, Huang G, Weng L, Xu J, Li Y, Cui W (2022). Decreased serum interleukin-41/IL-41 levels in patients with Graves' disease. J Clin Lab Anal.

[9] Cai X, Li K, Li M, Lu Y, Wu J, Qiu H (2023). Plasma interleukin-41 serves as a potential diagnostic biomarker for Kawasaki disease. Microvasc Res.

[10] Zhou Y, Shi S, Meng S, Zhao H, Wu X, Li M (2023). Potential clinical value of serum interleukin-41 levels in patients with acute gout. Int Immunopharmacol.

[11] Bridgewood C, Russell T, Weedon H, Baboolal T, Watad A, Sharif K (2019). The novel cytokine IL-41/IL-41 is elevated in Psoriatic Arthritis synovium and inducible from both entheseal and synovial fibroblasts. Clin Immunol.

[12] Gong L, Zhou Y, Shi S, Ying L, Li Y, Li M (2023). Increased serum IL-41 is associated with disease activity in rheumatoid arthritis. Clin Chim Acta.

[13] Zhang S, Lei Y, Sun T, Gao Z, Li Z, Shen H (2022). Elevated levels of IL-41 in rheumatoid arthritis: Association with disease activity. Cytokine.

[14] Kay J, Upchurch KS (2012). ACR/EULAR 2010 rheumatoid arthritis classification criteria. Rheumatology.

[15] Ushach I, Arrevillaga-Boni G, Heller GN, Pone E, Hernandez-Ruiz M, Catalan-Dibene J (2018). Meteorin-like/Meteorin-β Is a Novel Immunoregulatory Cytokine Associated with Inflammation. J Immunol.

[16] Singh JA (2022). Treatment Guidelines in Rheumatoid Arthritis. Rheum Dis Clin N Am.

[17] Hazlewood GS, Barnabe C, Tomlinson G, Marshall D, Devoe DJ, Bombardier C (2016). Methotrexate monotherapy and methotrexate combination therapy with traditional and biologic disease modifying anti-rheumatic drugs for rheumatoid arthritis: A network meta-analysis. Cochrane Musculoskeletal Group, editor. Cochrane Database Syst Rev.

[18] Chatzidionysiou K, Sfikakis PP (2019). Low rates of remission with methotrexate monotherapy in rheumatoid arthritis: review of randomised controlled trials could point towards a paradigm shift. RMD Open.

[19] Monserrat Sanz J, Bohórquez C, Gómez AM, Movasat A, Pérez A, Ruíz L (2020). Methrotexate Treatment Inmunomodulates Abnormal Cytokine Expression by T CD4 Lymphocytes Present in DMARD-Naïve Rheumatoid Arthritis Patients. Int J Mol Sci.

[20] Noack M, Miossec P (2019). Effects of Methotrexate Alone or Combined With Arthritis-Related Biotherapies in an in vitro Co-culture Model With Immune Cells and Synoviocytes. Front Immunol.

[21] Den Braanker H, Wervers K, Mus AMC, Bangoer PS, Davelaar N, Luime J (2020). Achieving sustained minimal disease activity with methotrexate in early interleukin 23-driven early psoriatic arthritis. RMD Open.

[22] Li H, Tsokos GC (2021). IL-23/IL-17 Axis in Inflammatory Rheumatic Diseases. Clin Rev Allergy Immunol.

[23] Slowikowski K, Nguyen HN, Noss EH, Simmons DP, Mizoguchi F, Watts GFM (2020). CUX1 and IκBζ (NFKBIZ) mediate the synergistic inflammatory response to TNF and IL-17A in stromal fibroblasts. Proc Natl Acad Sci.

[24] Shimamoto K, Ito T, Ozaki Y, Amuro H, Tanaka A, Nishizawa T (2013). Serum Interleukin 6 Before and After Therapy with Tocilizumab Is a Principal Biomarker in Patients with Rheumatoid Arthritis. J Rheumatol.

[25] Scherer HU, Häupl T, Burmester GR (2020). The etiology of rheumatoid arthritis. J Autoimmun.

[26] Ospelt C, Bang H, Feist E, Camici G, Keller S, Detert J (2017). Carbamylation of vimentin is inducible by smoking and represents an independent autoantigen in rheumatoid arthritis. Ann Rheum Dis.

[27] Van Wesemael TJ, Ajeganova S, Humphreys J, Terao C, Muhammad A, Symmons DPM (2016). Smoking is associated with the concurrent presence of multiple autoantibodies in rheumatoid arthritis rather than with anti-citrullinated protein antibodies per se: a multicenter cohort study. Arthritis Res Ther.

[28] Ponchel F, Duquenne L, Xie X, Corscadden D, Shuweihdi F, Mankia K (2022). Added value of multiple autoantibody testing for predicting progression to inflammatory arthritis in at-risk individuals. RMD Open.

[29] Maisha JA, El-Gabalawy HS, O'Neil LJ (2023). Modifiable risk factors linked to the development of rheumatoid arthritis: evidence, immunological mechanisms and prevention. Front Immunol.

[30] Al-tameemi S, Hameed N, Gomes K, Abid H (2022). Cigarette smoking increases plasma levels of IL-6 and TNF-α. Baghdad J Biochem Appl Biol Sci.

[31] Cuppen BVJ, Jacobs JWG, Ter Borg EJ, Marijnissen ACA, Bijlsma JWJ, Lafeber FPJG (2017). Necessity of TNF-alpha inhibitor discontinuation in rheumatoid arthritis is predicted by smoking and number of previously used biological DMARDs. Clin Exp Rheumatol.

[32] Cen T, Huang M, Li M (2024). Increased serum IL-41 associated with acute exacerbation of chronic obstructive pulmonary disease. Exp Ther Med.

